# Molecular Morphology of Telangiectatic Osteosarcoma Associated With Сystic Content: A Case Report

**DOI:** 10.7759/cureus.89658

**Published:** 2025-08-08

**Authors:** David Makaridze, Armaz Mariamidze, Tamuna Gvianishvili, Giulia Ottaviani, Liana Gogiashvili

**Affiliations:** 1 Histology and Embryology, David Tvildiani Medical University, Tbilisi, GEO; 2 Pathology, David Tvildiani Medical University, Tbilisi, GEO; 3 Clinical and Experimental Pathology, Ivane Javakhishvili Tbilisi State University, Alexandre Natishvili Institute of Morphology, Tbilisi, GEO; 4 Anatomic Pathology, Rossi Research Center, Università degli Studi di Milano, Milan, ITA

**Keywords:** aneurysmal bone cyst, diagnostic pitfalls, histopathology, immunohistochemical correlates, telangiectatic osteosarcoma

## Abstract

This case report is focused on a patient (a 32-year-old previously healthy man) with a femoral bone tissue tumor. The case underscores the diagnostic complexity of Telangiectatic Osteosarcoma (TOS) with Aneurysmal Bone Cystic (ABC) lesion and the importance of maintaining special immunohistochemical markers for differential diagnosis between low-grade osteosarcoma, primary ABC, and suspected TOS, which is a true mimicker of ABC. On the other hand, this presentation describes some molecular rearrangements seen in ABC.

## Introduction

Benign and malignant bone tissue tumors are often associated with aneurysmal or simple bone cysts. Among such tumors are Giant Cell Tumors, Fibrous Dysplasia, Telangiectatic Osteosarcoma, and Osteosarcoma. A literature review includes observations from some authors on over 130 cases in both pediatric and adult pathology. According to Papagelopoulos et al. (1998), ABC (Aneurysmal Bone Cyst) occurs in the population at a rate of 0.15 cases per million people per year [1].

In general, universal changes, different-sized spaces filled with blood, the presence of fluid-fluid levels, and cellular components are indicated, observed both in magnetic resonance imaging (MRI) and computed tomography (CT), and histologically confirmed as secondary ABC. Among them, the most prominent group of lesions is frequently described as primary ABC, osteosarcoma (including telangiectatic osteosarcoma), and fibrous dysplasia with aneurysmal contents [2].

Analysis of publications shows that specialists (pathologists, profile surgeons, and radiologists) are highly interested in the etiology of ABC, clinical features, and solutions for a wide range of cases: osteosarcoma, rhabdomyosarcoma, marker profiling, and radiological methods with assessments of their clinical value [3]. Attention remains focused on giant cell bone tumors, telangiectatic osteosarcoma (TOS), the postoperative state after orthopedic and trauma surgery, and chemotherapy as processes promoting ABC [4].

After studying the surgical-biopsy materials, tumors are often classified as ABC content tumors based on the "osteoblastic rimming" structure. Due to the increased frequency and complexity of the lesions mentioned above, the creation of a diagnostic algorithm was necessary as an alternative to primary preoperative biopsy. During the research, there was interest in the cause-and-effect relationship between the development and treatment of ABC and primary bone tumors, which necessitates the investigation of sensitive and specific markers. A direct correlation has been established between Ki67 expression and clinical-pathological and prognostic features of osteosarcoma, as well as other bone neoplasms, as confirmed by a meta-analysis of relevant studies (Ming Zeng, Jian Zhou, Lifang Wen et al., 2021) [5]. But there are the most relevant markers for testing of bone tumors, cystic tumors with novel correlates.

We present the observations on a bone tissue tumor case (TOS). The purpose of discussing this case is to evaluate the activity of cyclin-dependent kinase 4 (CDK-4) and murine double minute 2 (MDM2) expression as a molecular morphology marker of bone tumor formation versus other common immunohistochemistry (IHC) reactions. Some of these reactions were performed with immunohistochemical staining methods, but osteocalcin, MDM, CDK4, and p63 were analyzed under VentanaBench System protocols. The counting was done according to the following rule: The activity of markers was counted in the visual field of 100, including all cells except for inflammatory infiltrate cells (if such were present). In the changes similar to ABC, activity was counted in the following cells: osteoblasts, giant cells, and mononuclear cells, where the activity of Ki67 was 20% or more. Atypical mitotic figures were noted, and the resection margins were free. In the case of an osteosarcoma and ABC-like lesions combination, testing of Ki67 and MDM2 was also performed in multinucleated cells. The discussion of the presented case also serves as a comparative framework to identify and monitor similar cases in other regions and countries, which may be of broader clinical interest.

## Case presentation

A 32-year-old previously healthy man presented to the Department of Surgery at University Hospital “MediClub-Georgia” (Tbilisi) in February 2023 with a palpable, painful, and progressively enlarging mass located in the distal segment of the right femur. The changes had been present for approximately three months.

Histological specimens from operative materials, obtained after large femur amputation and curatage (an average of 25 specimens), were stained using hematoxylin and eosin (H&E). A panel of immunohistochemical and molecular markers was applied, including: 1) MDM2 -ventana antimouse monoclonal primary antibody; dilution 1:50/100, 4193 E3biquitine protein ligase; murine double minute 2 homolog (Human, Zitomed, Germany); 2) cyclin-dependent kinase-4 (CDK-4) - 12q14 ready to use also as cell division protein kinase-4 (Zitomed, Germany); 3) CD99 - Human recombinant, 1:100. Ewing sarcoma (EWS), synovial sarcoma (SS), low-grade fibromyxoid sarcoma marker (Leica Biosystems, USA); 4) p63 -transcriptional proteins group (p53-p73); 1:25, 7Jul mono. (Leica Biosystems, USA); 5) smooth muscle actin (SMA) L-CE; 1:100; (Leica, Biosystems, USA); 6) CD68 clone 514H12; mouse monoclon Anti-human L-CEH;1:25-1:100; 1 ml NCL-1-CD68; pH7,3; (Leica, Novocastra, USA); 7) Osteocalcin -osteoblast-osteoclast antibody interaction, 1:50; (Dako Systems); 8) Ki67 -1:100 (Santa Cruz Biotechnology, Santa Cruz, CA). The morphological component of the study, which included immunohistochemical and molecular marker analysis, was conducted at the Alexandre Natishvili Institute of Morphology, Tbilisi State University.

**Figure 1:**
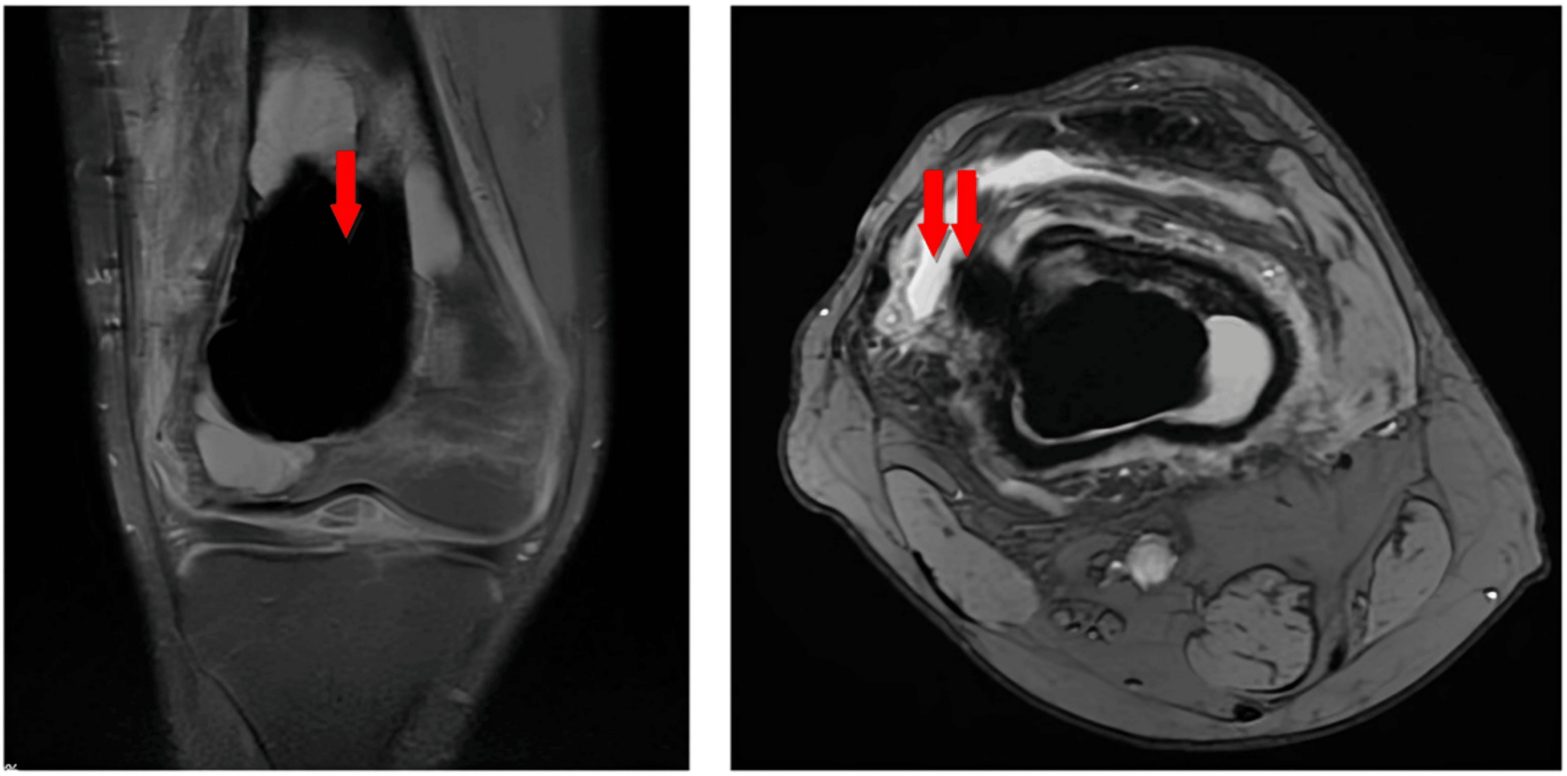
Preoperative MRI a. Sagittal section showing a lesion filled with fluid levels (arrow); b. Axial image showing a cystic-appearing lesion with mild surrounding soft tissue edema and irregular enhancement (arrows).

Radiological conclusions were provided for the observed patient, incorporating variables such as age, gender, tumor location, recurrence, and histopathological findings. Diagnosis was confirmed by three independent reference pathologists (DM, AM, and LG). Preoperative magnetic resonance imaging (MRI) revealed an irregular lesion with a thick, heterogeneously enhancing rim. Endosteal scalloping and periosteal reaction were noted (Figure 1).

**Figure 2:**
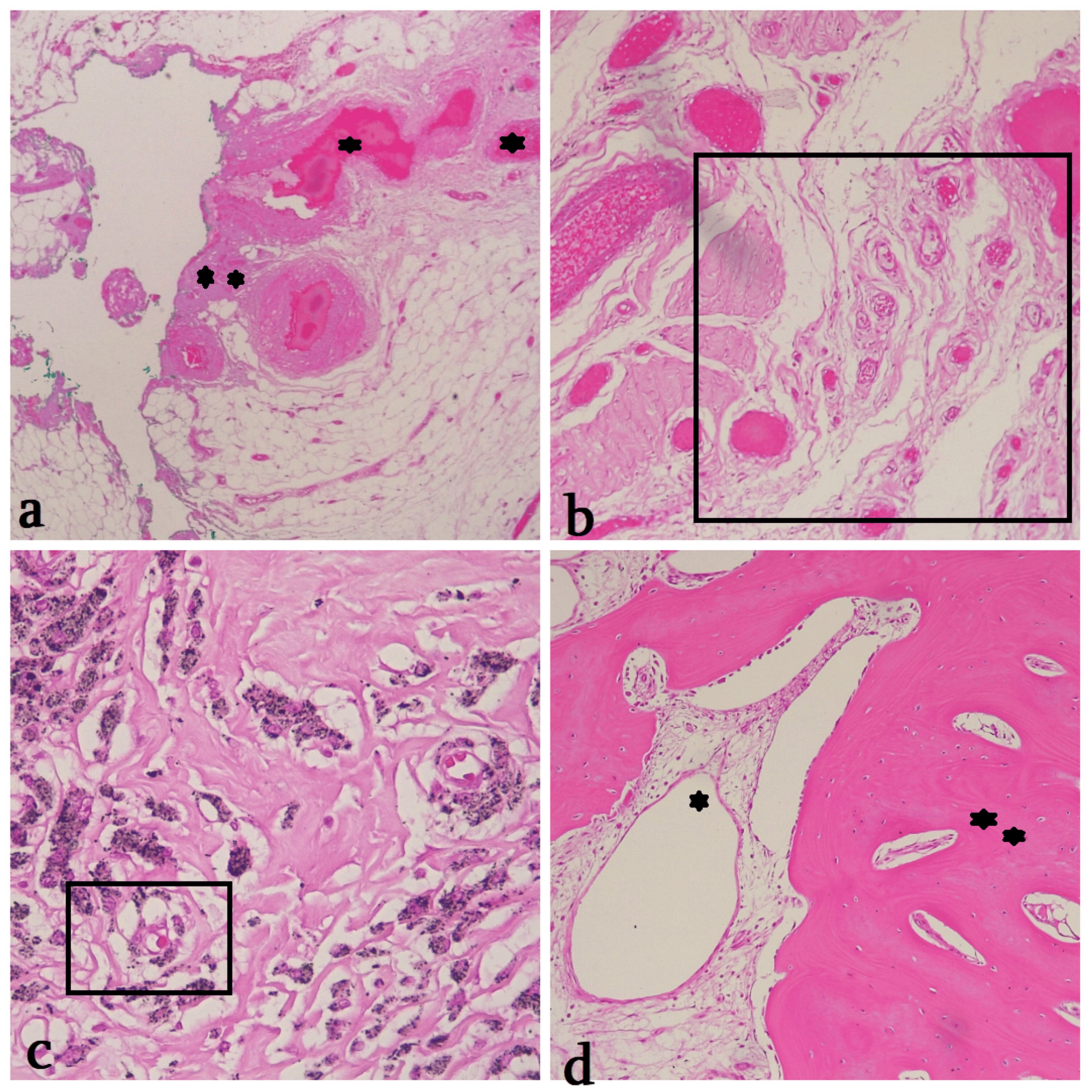
Telangiectatic osteosarcoma TOS: Telangiectatic osteosarcoma, H&E: Hematoxylin & Eosin stain. a. Aneurysmal Bone Cyst (ABC) like cystic lesion, with necrotic (*) and hemorrhagic area (**), 100X; b. Telangiectatic Sarcomatous fibrovascular foci in the tumor center (□), 100X; c. Solid proliferative malignant stromal cells with hemosiderin-laden macrophages (□), 200X; d. Osteogenic stroma (**) surrounding distorted bone cysts (*), 100X.

Surgical resection revealed a homogeneous, solid, black-grayish mass measuring 10 x 5,5 x 4,5 cm. The surface displayed cystic areas with fibrotic areas composed of solid proliferative giant cells and hemorrhagic cystic degeneration. In the background of osteoid material, giant cells with bizarre polymorphic nuclei, penetrating soft tissue, were observed. Histological examination after H&E staining showed: endosteal scalloping, a distinctive feature of aneurysmal bone cyst (ABC); multilaminated periosteal reaction, more typical of osteosarcoma; soft tissue involvement and matrix mineralization suggestive of malignancy. These findings indicated a malignant bone neoplasm with features of both stromal malignancy and ABC-like architecture (Figures 2a, 2b). Histological features confirm ABC content: necrotic and cystic areas filled with hemorrhagic material, hemosiderin-laden macrophages, numerous atypical giant cells, and spindle cell proliferation (Figures 2c, 2d). The tumor margin was within ≥ 1 mm of the resection line.

**Figure 3:**
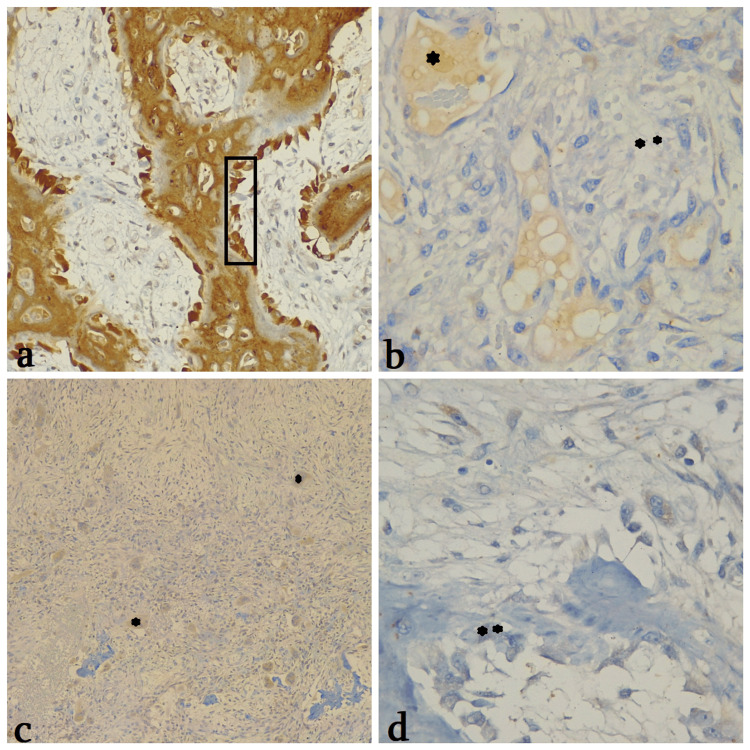
Telangiectatic osteosarcoma TOS: Telangiectatic osteosarcoma. Immunohistochemical results: a.Osteocalcin positivity in fibroblastic cells and osteoblastic “rimming” (□), X200; b. MDM2 heterogeneous perivascular positivity (*), negative stromal reaction (**), 400X; c. CDK4 positivity in multiple giant cells (*), 100X; d. p63 negative reaction in malignant cells (**), 400X.

Immunohistochemical results* came *positive for oteocalcin, MDM2, CDK4, SMA, CD68 (in mono- and multinuclear cells), and Ki-67 (20%) and negative for p63, CD99. Osteocalcin showed diffuse positivity in osteoblast "rimming" and fibroblastic tumor cells (Figure 3a), indicating preserved calcium homeostasis in metabolically active tissue. MDM2 expression was localized in perivascular tumor regions (Figure 3b), consistent with E3 ubiquitin ligase activity. CDK4 positivity was observed in numerous giant cells on a negative collagenous extracellular matrix background (Figures 3c), correlating with high Ki-67 proliferation and a negative p63 reaction (Figures 3d). These findings suggest dysregulated transcriptional control, possibly through disrupted ubiquitination pathways. Focally positive for CD68 (mono- and multinuclear cells), SMA, and cyclin D1. Immunohistochemical data confirmed diffuse positivity of giant cells for CDK4 on the negative collagenous extracellular matrix (Figures 3b, 3c), which correlates with Ki67 high (20%) activity and p63 negative reaction in malignant cells (Figure 3d). CD99 negativity helped exclude Ewing sarcoma.

Surgical management included femoral amputation with curettage and graft reconstruction. The patient underwent intensity-modulated radiation therapy and received three cycles of second-line and one cycle of third-line chemotherapy (doxorubicin). Unfortunately, despite treatment, the disease progressed with metastases to the lungs and brain. The patient died of tumor progression 12 months after initial diagnosis.

## Discussion

In this study, we present and compare a rare case of a bone tumor, telangiectatic osteosarcoma (TOS), with histological and molecular features resembling an aneurysmal bone cyst (ABC). The primary aim of this case report is to provide a detailed morphological and molecular description of TOS, highlighting its diagnostic challenges when ABC-like features are present. Reference is made to previously published studies by Kovac et al. (2023) [4] and Wadhwa N (2014) [6], which demonstrated that ABC-like lesions may not only arise from fibrous dysplasia but also share etiopathogenic links with trauma, hemodynamic alterations, and underlying bone pathology. Importantly, these studies recognize the potential for ABC-like changes to occur in association with osteosarcoma, including TOS.

The literature reviewed for this case was limited to English and Chinese publications, which may influence the generalizability and comprehensiveness of our findings [5,7]. Histologically, the neoplasm in our case originated from mesenchymal tissue and demonstrated distinctive membrane receptor activity. Immunohistochemical (IHC) analysis revealed relevant markers common to both ABC and TOS. The patient, a 32-year-old male, fits the demographic commonly reported in similar cases. Notably, MDM2 and p63 showed parallel expression patterns, suggesting deeper molecular interactions beyond superficial nuclear or cytoplasmic localization. These patterns may be related to deubiquitination processes affecting intracellular signaling pathways.

According to the 2020 WHO Classification of Bone Tumors, rearrangements involving the USP6 gene on chromosome 17p13 are typically observed in primary ABCs [8]. However, the presented case featured extensive bone destruction with a soft tissue mass, an atypical presentation for ABC but suggestive of malignancy, favoring a diagnosis of TOS with ABC-like morphology. The absence of USP6 rearrangement, coupled with MDM2 positivity and dysregulation of p53/p63 transcriptional activity, supports the diagnosis of TOS. Our findings are consistent with studies highlighting the significance of CDK4 and MDM2 in differentiating TOS from benign lesions [9]. CDK4 expression was observed in conjunction with a Ki-67 index of 20%, indicating increased proliferative activity.

Although some previously reported ABC cases share similar features, the distinguishing factors in our case include its secondary nature and association with benign fibro-osseous tissue. This rare combination contributes to the growing literature on borderline and malignant bone lesions in orthopedics and oncology. A systematic review of the literature using PubMed, Medline, and Mendeley databases [10] further emphasizes that osteolytic vascular lesions, often presumed benign (i.e., ABC), can obscure underlying malignant osteosarcoma (OS) components [11]. Particular attention should be paid to periosteal reaction and involvement of the epiphyseal plate when evaluating metaphyseal tumors [4,6].

Both MDM2 (a murine double-minute gene indicator) and CDK4 (cyclin-dependent kinase 4) were expressed in the tumor cells. MDM2, an E3 ubiquitin-protein ligase, has been proposed as a potential marker of malignant transformation in ABC-like lesions and may reflect underlying transcriptional dysregulation in OS. Its negative regulatory effect on p53/p63 activity strengthens the hypothesis of deubiquitinase involvement in TOS pathogenesis. We propose that the observed molecular patterns-particularly MDM2 overexpression, absence of USP6 rearrangement, and transcriptional suppression of p53/p63-may serve as a diagnostic pitfall. Such patterns could lead to misdiagnosis of TOS as ABC in cases with cystic architecture and limited osteoid formation. These insights are consistent with earlier findings [12], where MDM2 and CDK4 immunoreactivity aided in distinguishing low-grade osteosarcomas from benign fibrous dysplasia and TOS. Periosteal reaction and aggressive cortical destruction, as observed in this case, further support a malignant etiology. Several prior studies have reported cases initially diagnosed as ABC that were later identified as telangiectatic osteosarcoma [13]. Lechtholz-Zey et al. (2023) [14] also explored the USP6 rearrangement spectrum, highlighting its absence in malignant tumors with ABC-like morphology.

In conclusion, this case underscores the importance of a comprehensive immunohistochemical and molecular evaluation in distinguishing ABC from TOS. The presence of ABC-like features in osteosarcoma may obscure accurate diagnosis, but markers such as MDM2, CDK4, and the lack of USP6 rearrangement can provide critical diagnostic clues. Further studies are warranted to explore the implications of deubiquitination and transcriptional regulation in the pathogenesis and prognosis of telangiectatic osteosarcoma.

## Conclusions

The radiographic and histological features of telangiectatic osteosarcoma and cystic bone lesions, particularly aneurysmal bone cyst, are often difficult to distinguish. Although MRI can assist in diagnosis and is helpful for broadly excluding ABC, the presence of fluid-fluid levels is not pathognomonic for bone neoplasms, especially in TOS. However, multiple immunohistochemical studies have demonstrated clear differentiation between the lesion and the primary pathology, particularly through the detection of MDM2 and CDK4 rearrangements, as well as evidence of deubiquitination in cancer-associated pathways. Considering the increasing incidence of bone tissue tumors and their molecular-morphological significance, we believe that further research with more cases is needed.
